# Examining the social ecology of a bar-crawl: An exploratory pilot study

**DOI:** 10.1371/journal.pone.0185238

**Published:** 2017-09-27

**Authors:** John D. Clapp, Danielle R. Madden, Douglas D. Mooney, Kristin E. Dahlquist

**Affiliations:** 1 Suzanne Dworak-Peck School of Social Work, University of Southern California, Los Angeles, California, United States of America; 2 EarlyMoon LLP, Columbus, Ohio, United States of America; Waseda University, JAPAN

## Abstract

Many of the problems associated with alcohol occur after a single drinking event (e.g. drink driving, assault). These acute alcohol problems have a huge global impact and account for a large percentage of unintentional and intentional injuries in the world. Nonetheless, alcohol research and preventive interventions rarely focus on drinking at the event-level since drinking events are complex, dynamic, and methodologically challenging to observe. This exploratory study provides an example of how event-level data may be collected, analyzed, and interpreted. The drinking behavior of twenty undergraduate students enrolled at a large Midwestern public university was observed during a single bar crawl event that is organized by students annually. Alcohol use was monitored with transdermal alcohol devices coupled with ecological momentary assessments and geospatial data. “Small N, Big Data” studies have the potential to advance health behavior theory and to guide real-time interventions. However, such studies generate large amounts of within subject data that can be challenging to analyze and present. This study examined how to visually display event-level data and also explored the relationship between some basic indicators and alcohol consumption.

## Introduction

A fundamental fact underlying epidemiological indicators of drinking behavior and related problems is that all patterns and problems reflect either a single drinking event or an aggregate of drinking events. Acute alcohol problems have a huge global impact [1]; for instance, approximately 25% of all unintentional, and 10% of intentional injuries in the world can be attributed to drinking events. Drink driving, alcohol-related violence, and alcohol poisoning all occur at the event level. Despite this, alcohol research and tests of preventive interventions at the event level comprise a relatively small niche in the literature [2]. As noted by Clapp et al. [3], approaches to studying drinking events have advanced little over the past thirty years.

Conceptually, the social ecology of drinking events is complex and dynamic [3 4 5]. Systems dynamics models [4 5] based on field data have illustrated biological factors, (e.g., gender, body weight, etc.), motives, peer influence and the environment interact in complex feedback systems that influence intoxication (both peak blood alcohol content (BAC) and rate of BAC change). Although such models are useful to guide theory and pre-test potential interventions [6 7 8] validation and tuning of computational models with empirical data is critical [9].

Capturing the complexity of drinking events is methodologically challenging [2]. Historically, research into drinking behavior *in situ* has relied on retrospective survey methods, observation, or field interviews [10 11 12 13]. Beyond self-reports, many field studies of drinking have used breathalyzers to measure breath alcohol content(BrAC) and intoxication [14 15 16 17]. Although breathalyzers provide biological estimates of drinking that are arguably better than self-reports, the logistics of collecting breath tests in the field are difficult [18]. Further, with few exceptions [16 17 19 20 21] most studies using breathalyzers collect one sample per participant, making them cross-sectional.

Although point-estimates of BAC have utility (as do estimates of peak BAC), they are limited in providing useful data related to blood alcohol curves or how drinking shifts over the course of an event. Computational simulations of the dynamics of drinking events and the pharmacokinetics of BAC [4 5] strongly suggest that repeated measures of drinking during an event are needed to best understand BAC curves and the ecology of drinking events. Understanding the overall dynamics of drinking events and how BAC “behaves over time” is critical to identifying leverage points for intervention [22] and to avoid interventions based on simplistic models grounded in potentially spurious findings [23].

Transdermal alcohol monitors represent an alternative to breathalyzers, observation or self-reported drinking during drinking events [24]. While breathalyzers provide breath estimates of BAC (BrAC), transdermal alcohol monitors provide estimates based on alcohol perspired through the skin (transdermal alcohol content: TAC). One major potential advantage of using transdermal monitors over other methods is their capacity to take repeated TAC samples from the same subject over time. This feature has potential for enhancing event-level research, treatment outcome studies and the like. The proliferation of Global Positioning System (GPS) and Bluetooth equipped smartphones, smart phone applications and newer generations of smaller (wrist watch size) wearable alcohol or “tattoo” like monitors will likely improve our ability to study and intervene in alcohol events in real-time.

To date, however, there is only one known feasibility study that has been conducted using transdermal monitors to measure drinking during drinking events [25]. Otherwise, event-level studies are still failing to include more continuous objective measures of alcohol consumption. There are a handful of studies that have explored the use of transdermal sensors in contingency management interventions but behaviors are followed more aggregately [26 27 28]. The devices are more typically utilized as an intervention in a criminal justice setting to decrease the propensity of reoccurring harm such as drink driving [29]. Although recent advances in data collection technologies [30 31] have the potential to advance our understanding of event-level drinking behavior, Riley et al. [30] notes that our ability to collect individualized, context-specific data and to intervene *in situ* has surpassed our current theories. The authors note that “health behavior models that have dynamic, regulatory system components to guide rapid intervention adaptation based on the individual’s current and past behavior and situational context” are greatly needed.

This exploratory pilot study sought to examine event-level drinking using transdermal monitors, coupled with ecological momentary assessments and geospatial data. The goals of the study included 1) examining how to visually display event-level data that are dynamic, 2) identifying data mining and analyses methods to handle complex “big data, small N” [32] data, and 3) to model some basic alcohol indicators for this drinking event (a senior bar crawl).

## Methods

This study was a prospective, repeated measures investigation of within-night drinking during one bar crawl event that occurs yearly near graduation at a large Midwestern state university in the U.S. This study included retrospective surveys of past drinking behavior and prospective examinations of a drinking event including real-time alcohol monitoring coupled with geospatial data.

### Sample

Twenty undergraduate students (*n* = 20) enrolled at The Ohio State University were recruited to participate in this study. Participants were active consumers of alcohol (i.e., self-reported alcohol use at least once during the preceding week), legally able to drink (i.e., 21 years of age or older), and owned a smartphone (it was a required instrument for survey administration). All participants planned to participate in the “senior” bar crawl during spring semester 2016. This bar crawl occurs yearly near the end of spring semester in a particularly dense bar district directly across from main academic center of the campus.

### Procedure

This study was approved by The Ohio State University Institutional Review Board (2016B0092). Students were recruited to participate via flyers and college newsletter announcements. Participants attended an orientation meeting at our research office prior to starting the bar crawl where verbal informed consent was obtained. Each participant was insured that they could opt out of the study at any point. During this meeting, participants completed baseline survey measures on a secure online survey platform that assessed demographics and past alcohol use behaviors. Each participant was then fitted with a SCRAM-CAM transdermal device in order to continuously assess transdermal alcohol content over a 24 hour period. More information about this transdermal device is provided below. After the meeting, participants were told to attend the bar crawl as they originally intended. We did not encourage the participants to drink more or less than they otherwise would and they were not required to attend any specific bar or location during the bar crawl.

During the crawl and throughout the rest of the afternoon and evening, participants responded to ecological momentary assessments (EMA) accessed on the web and housed on the survey platform Qualtrics. EMAs are utilized to repeatedly report on behaviors as the experience is occurring [33] and are a feasible approach to assess drinking events *in situ* with minimized recall bias. In this study, EMA surveys were delivered once hourly from 12pm to 5pm and again at 9pm and 12am. Participants were provided with a link to each survey via text message. A reminder to complete the survey was sent 5 minutes passed the hour. Participants provided information about their current alcohol consumption, drinking location, drinking companions, and subjective intoxication. Additionally, the respondent’s geographical location was logged with each survey response.

During the next day, participants returned to our office to remove the transdermal device and to respond to a short survey about their experiences at the bar crawl. Participants reflected whether or not they had fun, met new friends, lost track of time, or experienced any alcohol-related consequences (i.e., physical injury, hangover). Additionally, participants reflected on their motivations throughout the evening, particularly whether they drank more than they intended. Participants received $50 as an incentive for their participation.

### Measures and instruments

This study included a wide variety of data and many aspects of the drinking event were followed. Only the measurements that were found to be important are included in this description. The full instruments utilized in this study are available from the corresponding author.

#### Demographics

Participant gender, age, weight, ethnicity, living situation (i.e., on-campus or off-campus), employment status, major, grade point average, membership in Greek organizations or on athletic teams were assessed. These items have been shown to be predictive of heavy drinking in past empirical work [34].

#### Historical alcohol use

Participant alcohol use history was assessed with both the Alcohol Use Disorders Identification Test (AUDIT) [35] and quantity and frequency measures adapted from the National Epidemiologic Survey on Alcohol and Related Conditions (NESARC) [36]. The AUDIT identifies harmful patterns of alcohol consumption with 10 items that are summed to create an overall score. Scores of 8 or greater point to a possible alcohol-use disorder, scores of 8–15 indicate hazardous alcohol use, scores of 16–19 represent harmful use, and a score of 20 or more is indicative of dependence. In addition to the possibility of an alcohol-use disorder, overall drinking rates were assessed as well as the consumption of specific types of beverages (e.g., beer, wine, or liquor) in a Quantity/ Frequency (“QF”) measure. Participants were asked how often they consumed each type of alcohol or how often they felt drunk in the past 30 days on a 7-point Likert scale from (1) every day to (7) once a month. Items from NESARC were altered to reflect the frequency and amount of alcohol each participant consumed over the past 30 days instead of the typical 12 month timeframe. Reflecting on alcohol use during the past month enlists more accurate recall than a 12 month period [37].

#### Pre-drinking plans (“Plans”)

Before participants attend the bar crawl, they reported their plans for the evening. Specifically, the amount of money they planned to spend, the amount they intended to drink (i.e., not enough to get buzzed, enough to feel a slight buzz, enough to feel a little drunk, or enough to feel very drunk), the number of places they intended to go, where they intended to drink (i.e, only bars on the crawl list, other bars, friend’s apartments) and the mode of transportation they planned to take (i.e., walk, bike, ride in a car, drive, take a bus or taxi) was assessed. Participants also commented on whether they planned to play drinking games or whether they could access illegal drugs if they wanted.

#### Ecological momentary assessments

Surveys administered during the bar crawl assessed participant activity hourly. Participants recorded the number and type (i.e., beer, wine, liquor) of drinks they consumed during the past hour as well as the amount of money they spent (in U.S. dollars). Participants were reminded of the standard U.S. drink size for various types of beverages with a drawn depiction every time the amount of alcohol one consumed was requested. Current subjective intoxication was analyzed as well (i.e., whether the participant felt no buzz, slight buzz, a little drunk, or very drunk). This “Feel Now” single-item measure has been utilized to measure one’s perceived level of intoxication in a number of field studies to date [38 39 40 41]. Each EMA response captured geospatial (GPS) data gathered from the participant’s cellphone so it was possible to determine the approximate location of the participant when the survey was completed. The total number of drinks the participant self-reported consuming (based on each individual EMA survey response) was summed for the entire drinking event. The total amount of money spent was also calculated for the entire event.

#### Next morning (“Post”)

Participants completed survey items that reviewed the events during the preceding day. They specified the number of bars in which they consumed alcohol during the course of the entire drinking event, whether they perceived time to move more quickly than usual, and if they consumed more alcohol than they originally intended. Participants additionally estimated at what time they consumed their first drink and when they finished their last drink of alcohol.

#### Transdermal alcohol sensor (SCRAM-CAM)

The Secure Continuous Remote Alcohol Monitoring sensor (SCRAM-CAM), developed by Alcohol Monitoring Systems, Inc., can detect ethanol concentration in vapors formed above the skin. The bracelet is fastened to the participant’s ankle and cannot be removed until the device is unlocked. Every 30 minutes, the sensor conducts a reading of transdermal alcohol. Information is stored in the device and can be uploaded to a computer application after the device is removed. Alcohol that is detected transdermally corresponds well with blood or breath alcohol concentrations [42 43]. The SCRAM-CAM is commonly utilized to monitor individuals on house arrest for alcohol-related offenses. More recently, this device and comparable sensors have been utilized in research and have become a more common method to test alcohol use [25]. While these devices are not regularly utilized in studies among college students as of yet, a few recent attempts have illustrated the inherent benefits of a noninvasive and continual method for estimating blood alcohol content (BAC) [25 27 31]. Both the research participant and the research team are blind to the BAC readings during the observation period.

### Analysis

Initially, descriptive data for participant demographics were explored in Python 3.5.2. Next, the ability to visualize event-level data with multiple collection techniques (i.e., continuous transdermal data, hourly surveys, and spatial-temporal data) was tested with Python’s Matplotlib 1.5.1 package of visualization and plotting tools. Lastly, exploratory analyses (or data mining) were run in order to identify relationships that might be of interest for further study including more proper assessment of test validity. Simple Linear Regression, Boxplots and ANOVA analyses, and mixed-effects regression models were utilized to assess data in the exploratory analyses. These models were run in *R* using the nmle package. Relationships with p-values of 0.05 or smaller are discussed in the results.

For the Exploratory Data Analysis, four dependent variables were examined: 1) peak transdermal alcohol concentration (TAC) value; 2) average rate of TAC change prior to peak; 3) maximum rate of change of TAC prior to peak; and 4) time to peak TAC value. The peak TAC value was provided in the output from the manufacturer. The average rate of TAC change prior to peak was determined by taking the ratio of the peak TAC value to the time required to reach that value from the adjusted start of drinking (the earlier of when the participant claimed to start drinking via self-report and the first consistent non-zero TAC value). The maximum rate of change of TAC prior to the peak TAC value was computed as the maximum derivative of the TAC curve over this period as given by the Savitzky-Golay [44] filter (described below). The time to peak TAC was determined based on both self-reported time spent drinking and the TAC measurements. In a few cases, a consistent non-zero TAC level began to be recorded before the participant self-reported drinking. For the purpose of this metric the time to peak TAC represented the time from the earlier of self-reported drinking time and the beginning of consistently non-zero TAC measurements.

#### Assessing transdermal data

According to the manufacturer there is an approximate 45-minute lag between alcohol entering the blood stream and the SCRAM device reporting that it observes that alcohol through sweat. Due to this lag, all TAC readings were shifted 45 minutes forward in time. This enables the comparison of reported drinking activity with changes in TAC. Though discrepancies may exist based on individual differences in skin thickness for instance, we believe 45 minutes is enough to account for the lag in metabolizing.

The TAC data series were noisy, which is to be expected with sensor readings. A smoothed signal was obtained using the Savitzky-Golay filter [44], a standard filter for removing noise from time series. The Savitzky-Golay filter has two parameters that need to be set. One is a data window size and the smoothed estimate is computed from the points within the window. The other is a smoothing parameter which can range from zero to N-1 where N is the number of data points in the window. When the smoothing parameter is set to zero, the Savitzky-Golay filter is simply a moving average filter that estimates the signal value at a point by averaging all the data values in the window about the point. If the parameter is set to one, then the Savitzky-Golay fits a line to the points in the window and estimates the signal using this line. Each point has a different set of points in the window and hence each point is estimated with a different line. Higher parameter values enable the filter to fit quadratics, cubic, etc. based on the points in the window. At the extreme value of N-1, the fit polynomial is an interpolation function that passes through each of data points. In this case, the estimated value is just the original value with no smoothing. Thus the Savitzky-Golay filter’s smoothing parameter provides a range of smoothing from a Moving Average to none at all. Also, the window size impacts the degree of smoothing with larger windows smoothing more and in the process damping peaks. Thus, the filter needs to be tuned to the data. By examining a range of parameters for the TAC data and examining residuals, a window of size 15 and a smoothing parameter of 5 were determined to eliminate the high frequency movements of the signal, while preserving the slower changing TAC signal.

The Savitzky-Golay filter can also be used to provide the derivative of the signal. In the case of TAC data, it represents the rate of change in TAC at any point in time. The data from the SCRAM devices were not necessarily collected at the same time point that as survey was completed. Cubic-spline interpolation of the Savitzky-Golay filtered TAC data was used to estimate both TAC and rate of TAC levels at points in time where no TAC was taken.

#### Device malfunctions

The company that manufactures SCRAM reviewed the TAC data for each subject. The ankle of one of these subjects was too large for the SCRAM device to work properly (skin contact was not adequately maintained) and these data were removed from the study. Another participant’s data did not pass a test of tampering with the device, perhaps due to alcohol spilled on the sensor or the presence of a foreign object that blocked readings and this subject’s TAC data were also removed. The resulting data set resulted in a sample of eighteen (*n* = 18) participants with TAC data series through their peak TAC value and for some period afterward. However, it should be noted that half of the remaining sample had some erratic TAC readings that required smoothing. A total of nine subjects had one or more data points that were either zero or very low between two substantial TAC measurements. Specifically, two participants showed evidence of alcohol spillage or otherwise unreliable data from some point after their peak TAC measurement was observed. These data were truncated and the first part of the data series were retained and used. Two other subjects had erratic data and although the company cautioned about the use of this data, their data were retained after examination. The use of smoothing algorithm, explained below, helped to mitigate the noise. Data points at or near zero were judged as non-physical measurements as they were inconsistent with the rate that the body processes alcohol and hence almost surely due to a sensor reading malfunction. These data were modified to remove the TAC measurement at or near zero between two substantial nonzero values.

#### Missing data

Two participants had missing GPS data. These two were retained for all analysis except the spatial analysis. The spatial analysis also used TAC readings. For this analysis the two participants who were removed from the TAC analysis were also removed from the GPSdata set for *n* = 16.

## Results

### Demographics

Of the 20 participants, 10 were male and 10 were female. There was little variation in age with all participants having ages in the range of 21 to 23 years old. Seventy percent (14) identified themselves as White while the remaining 30 percent identified themselves as Asian. There were three Social Work majors, three Neuroscience majors, and two Nursing majors. The remainder of the students had different majors. Five were in the Greek system and two were athletes. One person lived on campus, the remainder lived near campus in either apartments (12) or houses (7). The participants’ GPAs ranged from 2.65 to 3.81 with half of the participants earning a 3.50 or better. Weights ranged from 115 pounds to 320 pounds with median and mean weight of 157.5 and 167.7 pounds respectively.

### Data visualization

#### Baseball card plots

**Fig 1:**
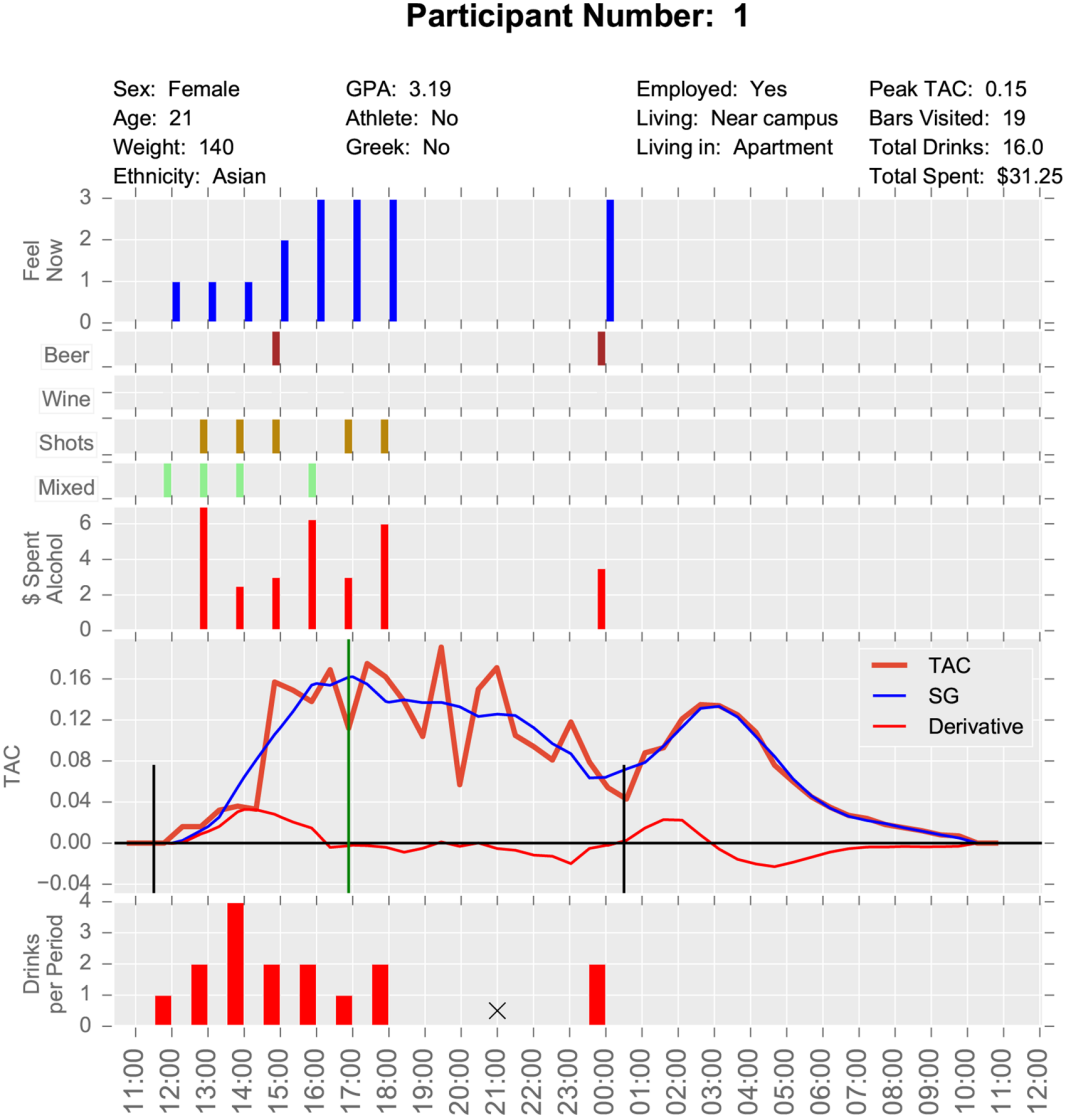
Baseball card plot for Participant 1.

**Fig 2:**
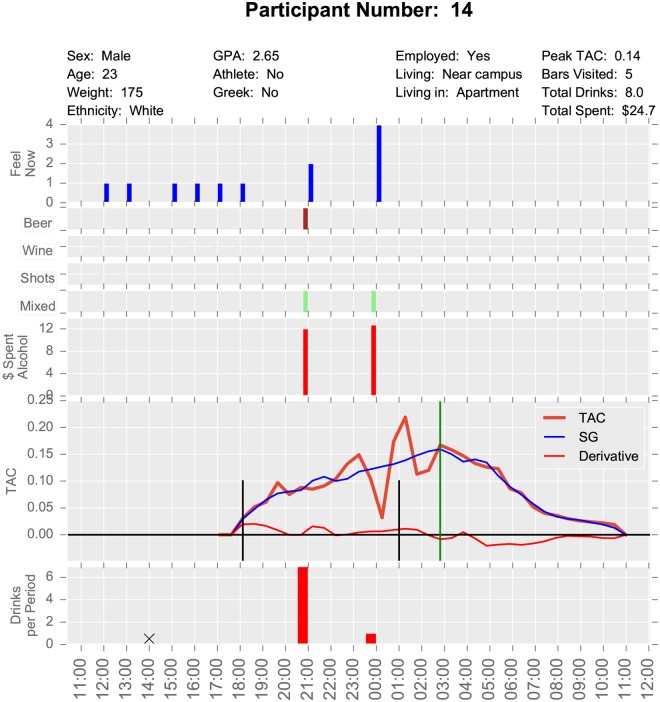
Baseball card plot for Participant 14.

This study had multiple data sources. TAC sensor data was collected every half hour, survey data was collected at varying intervals during the bar crawl event, and pre- and post-event surveys were also completed. For this project, we used what we term “baseball card plots”, which layout basic demographics and metrics for a participant on a single sheet of paper, not unlike the information about a ball player on a baseball card. Examples are seen in Figs 1 and 2.

The top plot of these visualizations contains time independent data such as demographics and event level statistics including the peak TAC reached, the number of bars visited, the total number of reported drinks, and the reported total amount of money spent on alcohol. Time series data are reported down the page with all graphs having a common time axis on the bottom. The bottom plot records the self-reported number of drinks at each survey time. The plot bar markers have their right edge on the survey time as they reflect behavior over the period since the previous survey. A black X indicates that a survey was scheduled for that time but not completed (i.e., missing).

The next plot up gives the TAC readings as a function of time. These curves represent the data after being shifted to the left by 45 minutes due to the lag between alcohol entering the blood and its measurement in sweat by the TAC device. This plot shows the raw data, the Savitzky-Golay smoothed data, and the Savitzky-Golay derivative which gives the rate of change of TAC. The vertical black lines indicate the reported beginning and end of drinking and the green vertical line is located at the peak TAC value. Above the TAC plot is the self-reported amount of money spent on alcohol since the previous survey.

The next four plots represent the type of alcohol consumed since the previous survey. These are yes or no responses. Participants can report multiple types of alcohol in a survey period, but we did not capture the number of drinks of each type. The top plot is a self-reported rating of how intoxicated the participant feels (referred to as the “Feel Now” variable). The responses of 1 to 4 correspond respectively to (1) not buzzed, (2) slight buzz, (3) a little drunk, and (4) very drunk. This plot enables us to compare self-reported drinking types, amounts, and times with TAC and self-perceived intoxication.

These two participants had similar peak TAC values, but different patterns of consumption. Participant 1 began consuming at 11:30 am and drank until after midnight. She visited 19 bars and reported having 16 drinks. She missed the 9:00 pm survey so there is uncertainty about whether she actually consumed a total of 16 drinks. Her pattern was to drink the most heavily in the midafternoon with 4 drinks in the one hour between 1:00 pm and 2:00 pm. She reached her peak TAC at about 5:00 pm. Participant 1 drank a mixture of beer, shots, and mixed drinks. We observe a second peak in TAC value at about 3:00 am, corresponding to a couple drinks at midnight. By contrast Participant 14 did not begin drinking until roughly 6:00 pm and stopped drinking at 1:00 am. He visited 5 bars and drank 8 drinks, which consisted of mixed drinks and beer. He consumed most of his alcohol between 6:00 pm and 9:00 pm with only two drinks after 9:00 pm. We note that both participants’ self-reported “Feel Now” (i.e., their subjective intoxication) score tracked well with their actual TAC levels.

#### Spatial-temporal movement

**Fig 3:**
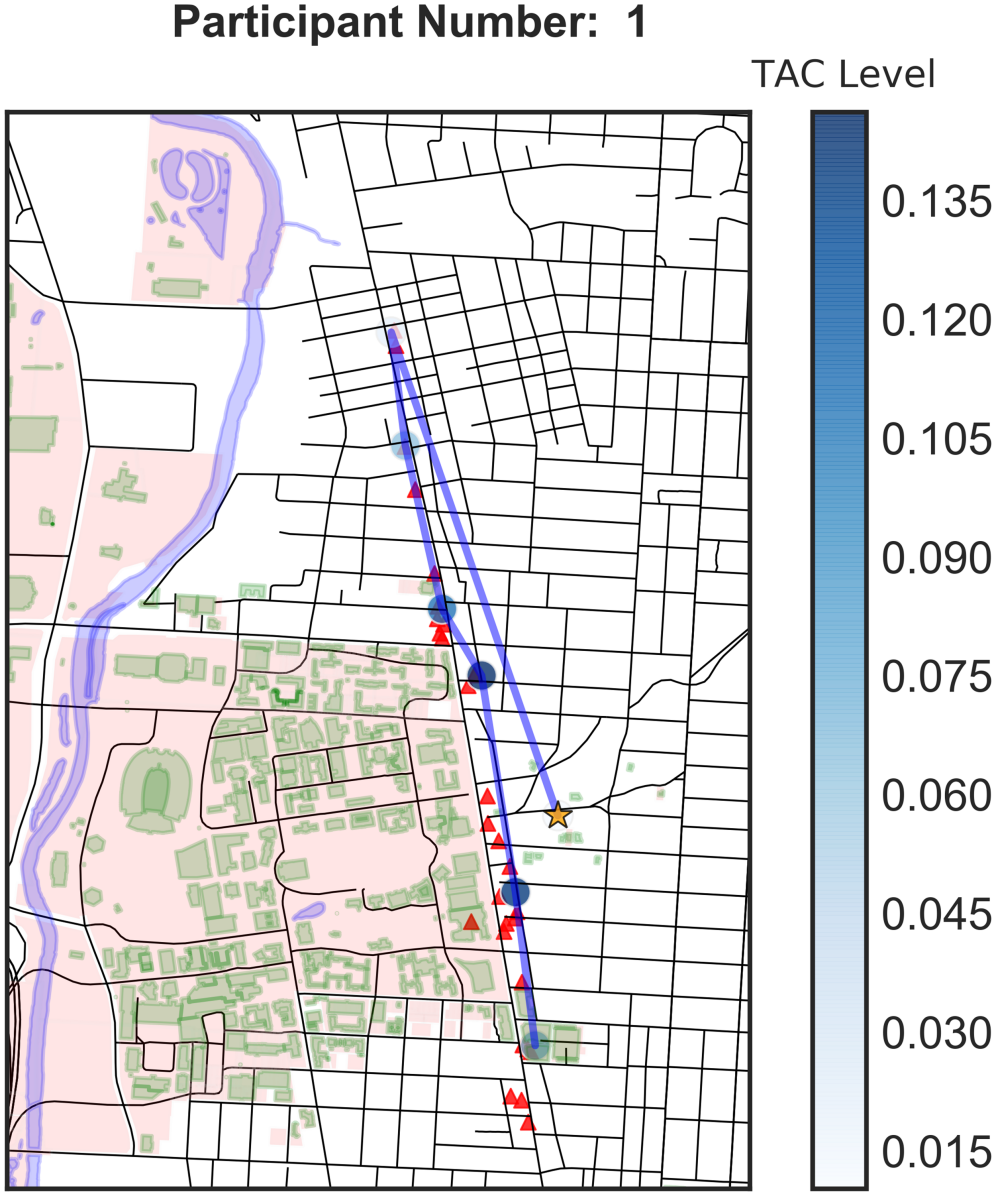
Spatial plot for Participant 1.

**Fig 4:**
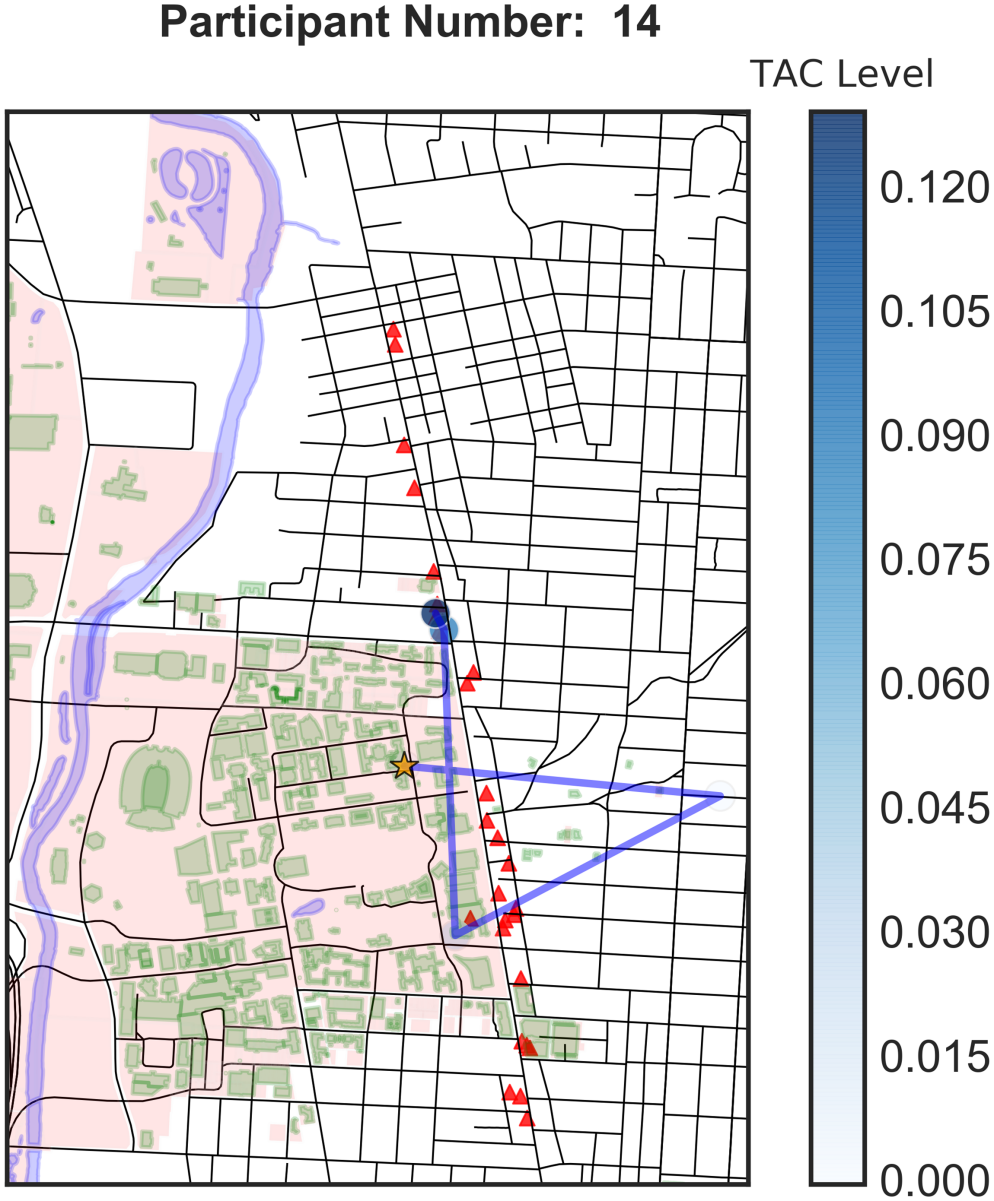
Spatial plot for Participant 14.

**Fig 5:**
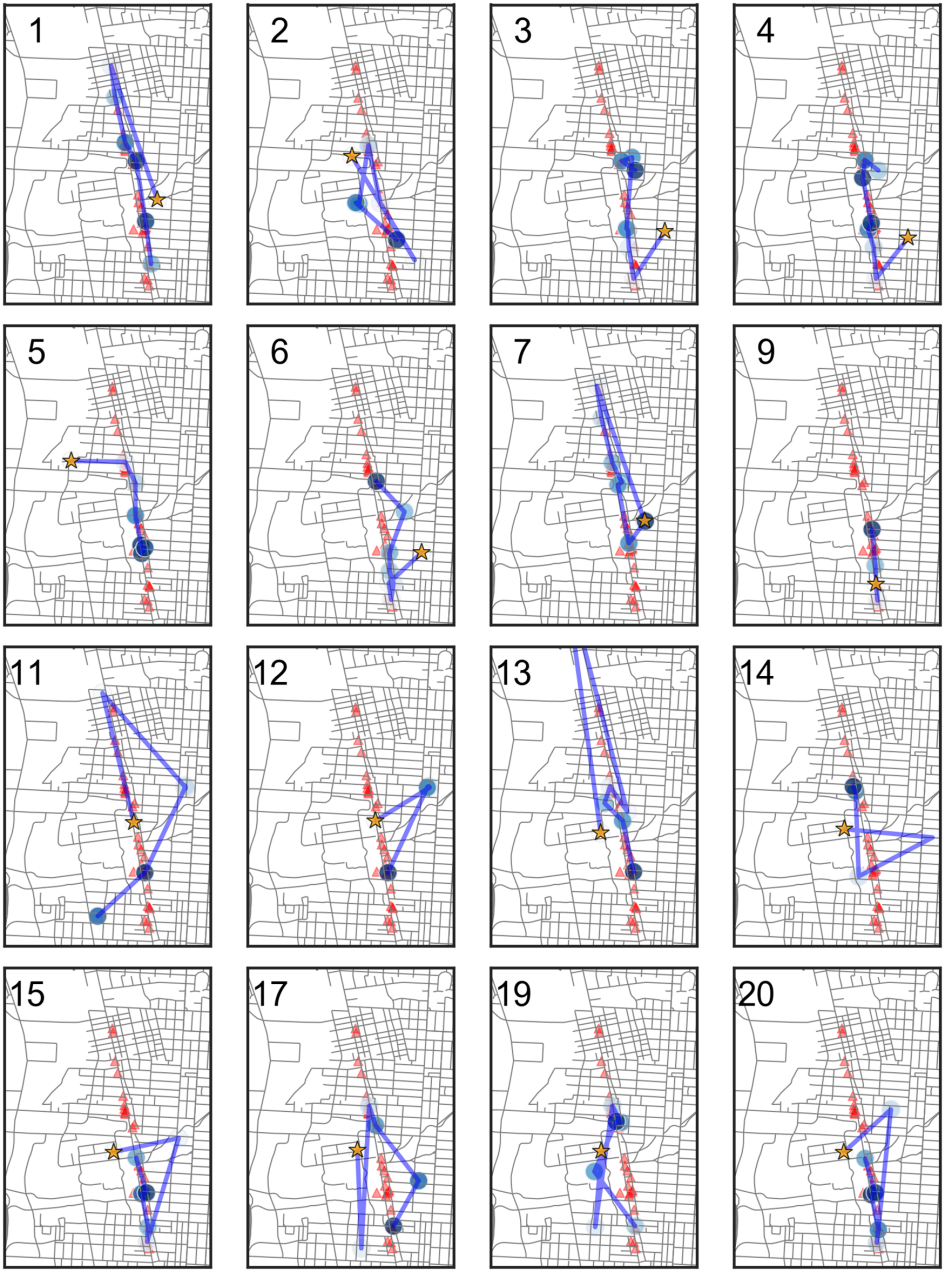
Spatial plots of pilot participants.

The use of GPS devices allows us to understand how participants move throughout the bar crawl event. Fig 3 shows the movement of Participant 1. The campus area is shaded in pink with its buildings in green. The red triangles indicate the local bars participating in the bar crawl. The orange star shows that Participant 1 began the event off-campus, moved to the northernmost participating bar and then traversed the strip. She did not continue to the southernmost bars. The blue circles indicate survey periods and the shade of blue indicates the TAC value at the time of the survey. Here we observe that she had a higher blood alcohol level during the middle of the event and had a lower level by the time she was attending the last bars. This pattern is supported by her baseball card plot (Fig 1). Fig 4 shows the spatial plot for Participant 14. He started from an on-campus building and then went off-campus, possibly home. Then he went to an on-campus bar in the student union and went north and ended the evening at bars north of campus. Fig 5 shows the spatial trajectories of all 16 participants for which we have both GPS and TAC data. We observe a variety of patterns of bar visitation ranging from covering the length of the bar strip to concentration on a portion of the strip.

### Exploratory findings

#### Relationship between four main TAC dependent variables

**Fig 6:**
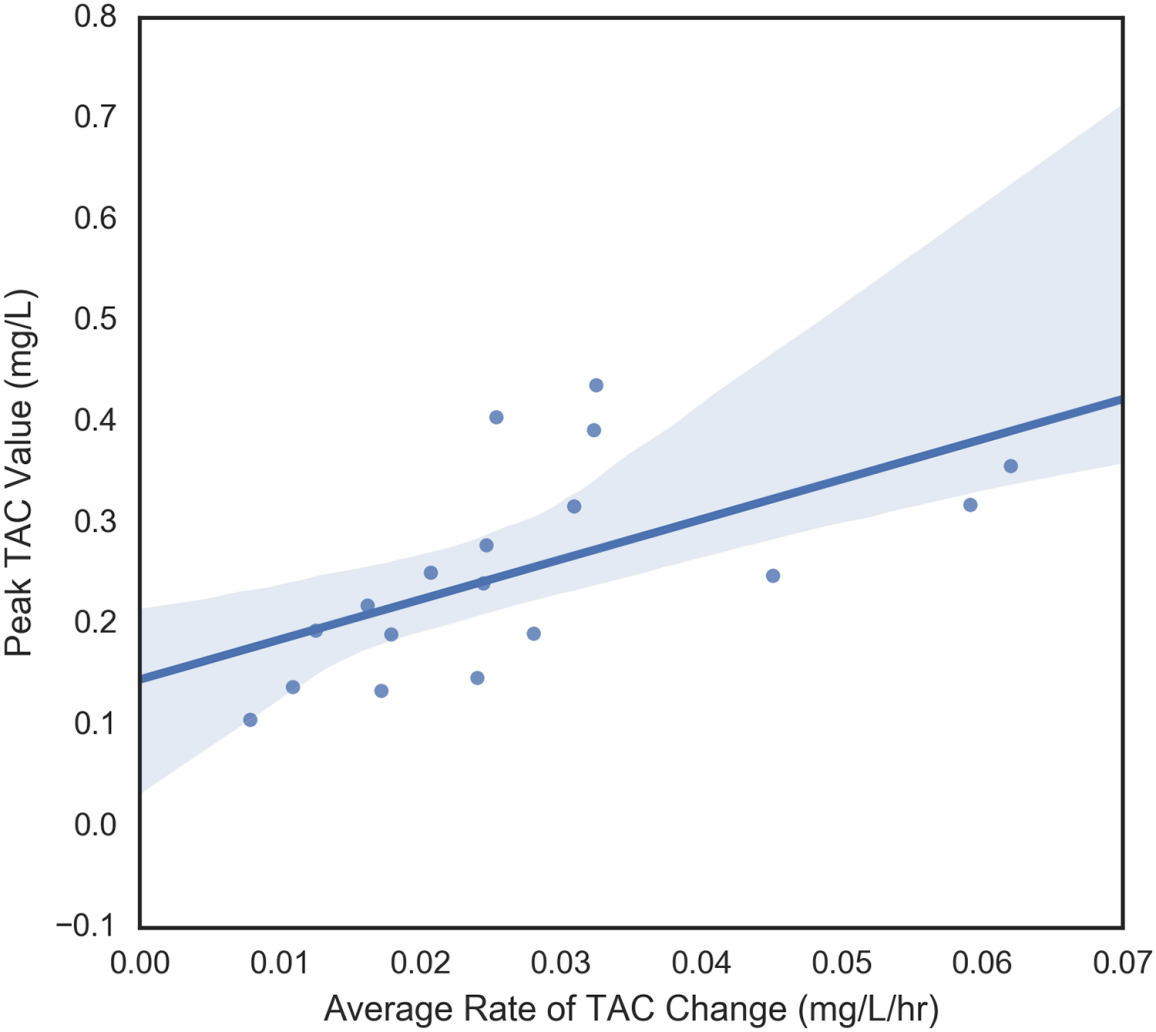
Average rate of TAC vs peak TAC.

**Fig 7:**
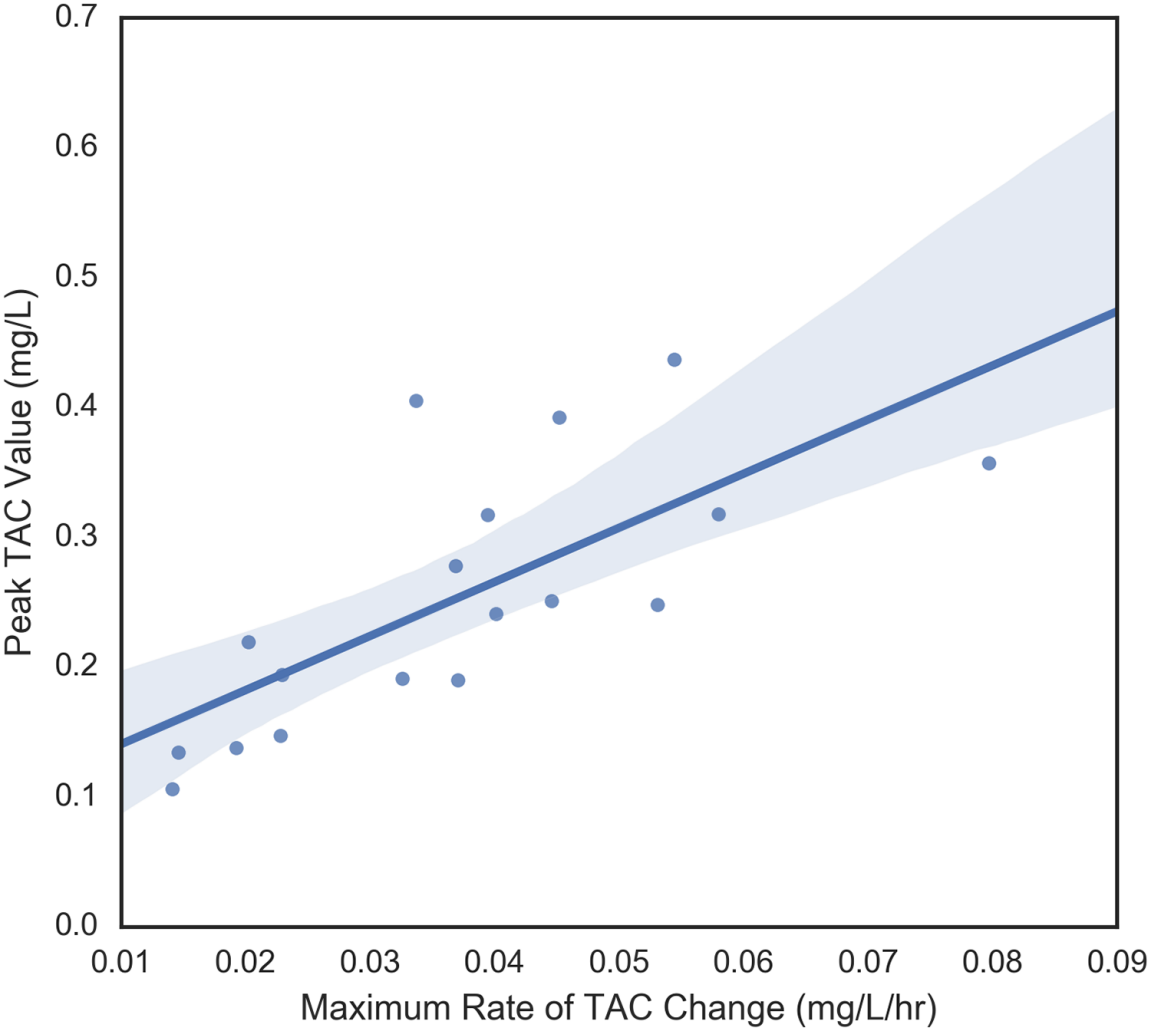
Maximum rate of TAC vs peak TAC.

**Fig 8:**
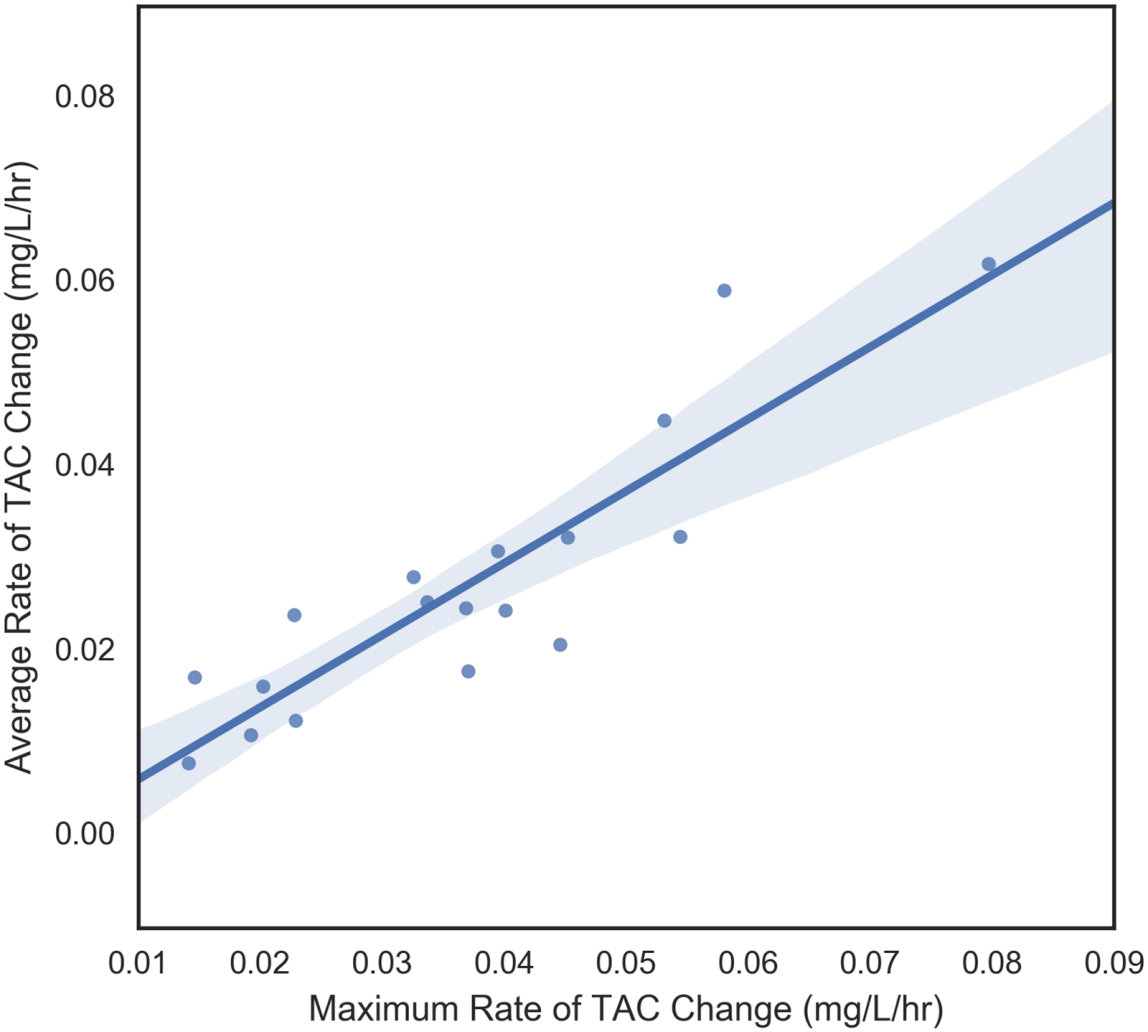
Maximum TAC rate vs average TAC rate.

The four dependent variables that were examined were peak TAC, average rate of TAC change prior to peak, maximum rate of change of TAC prior to peak, and time to peak TAC. These variables measure different aspects of blood alcohol concentration so it makes sense to begin by understanding their relationships with each other. These pair-wise relationships are shown in Figs 6 through 11. It would be expected that more rapid consumption of alcohol leads to higher peak blood alcohol levels and this is supported in these data. Figs 6 and 7 show the relationship between the peak TAC value and the average and maximum rate of TAC increase, respectively. The simple linear regression models for both were significant (p = 0.0087 and p = 0.00078) and explained 35.8 and 51.6 percent of the variance in the peak TAC value, respectively. Fig 8 addresses the relationship between maximum rate of TAC increase and average rate of TAC. As both are ways of measuring rate of consumption, it is expected that these variables are highly correlated. A simple regression model of the relationship was highly significant (p = 6.32e-07) with an R-square of 0.797.

**Fig 9:**
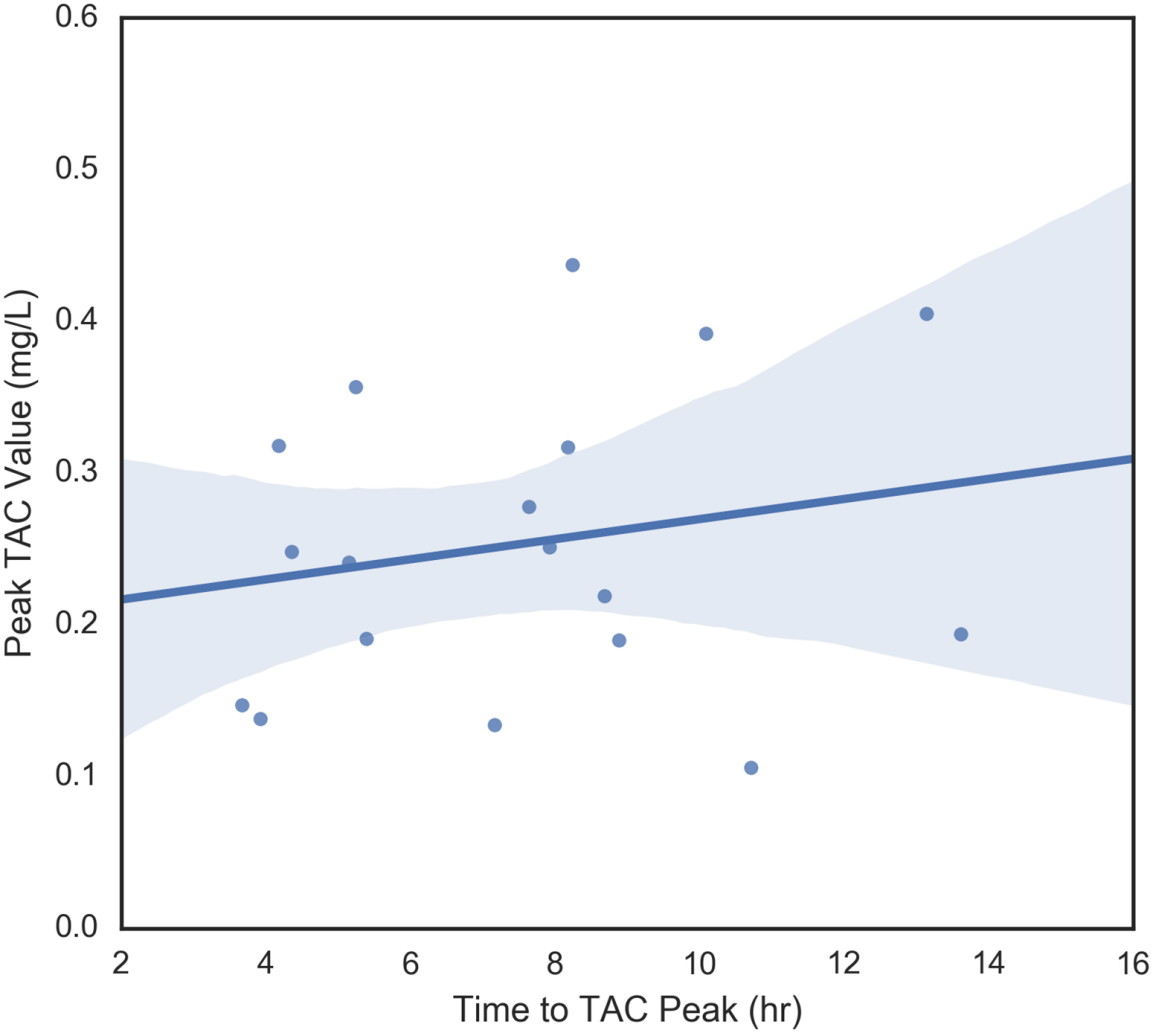
Time to TAC peak vs peak TAC.

**Fig 10:**
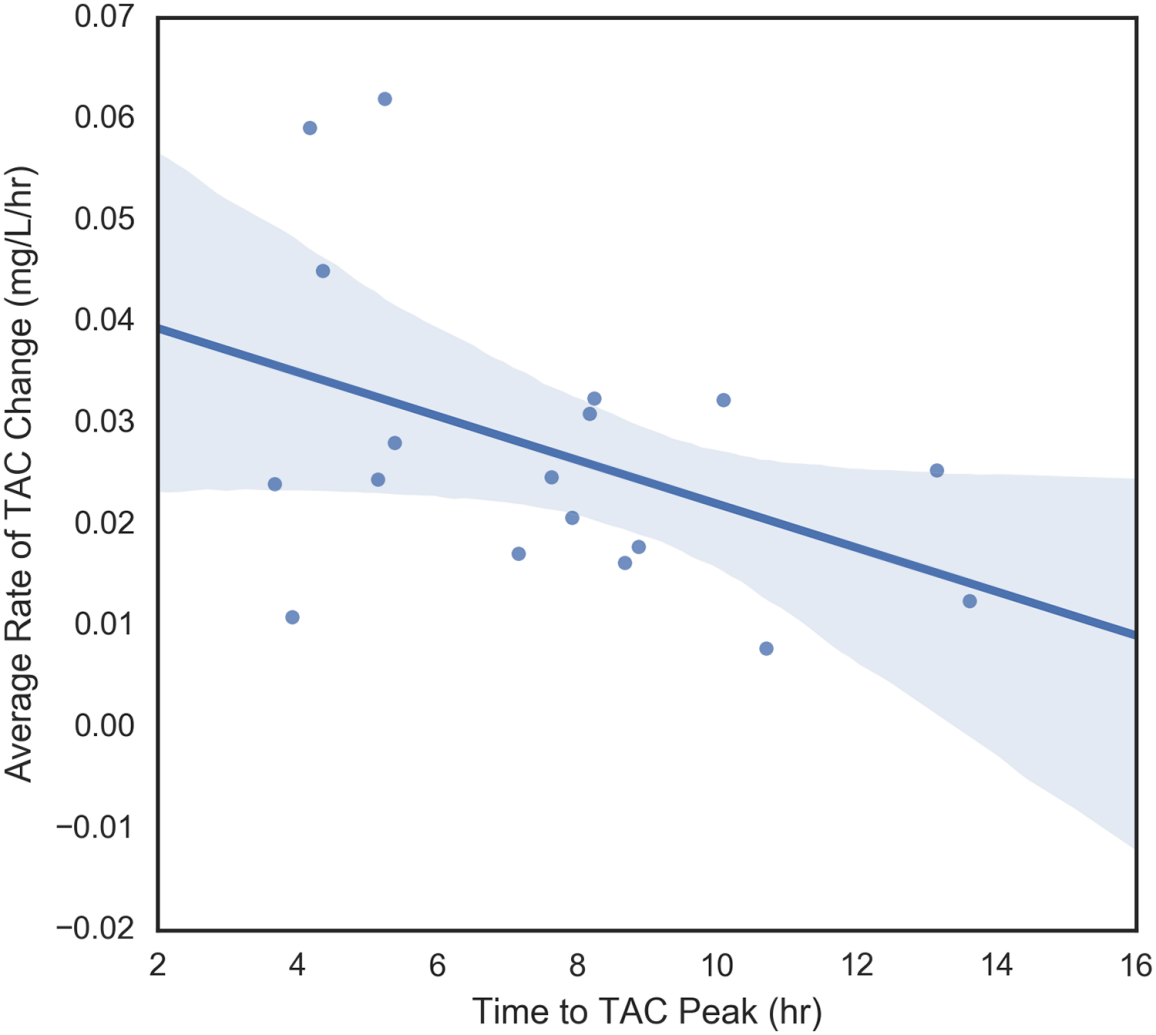
Time to TAC peak vs average TAC rate.

**Fig 11:**
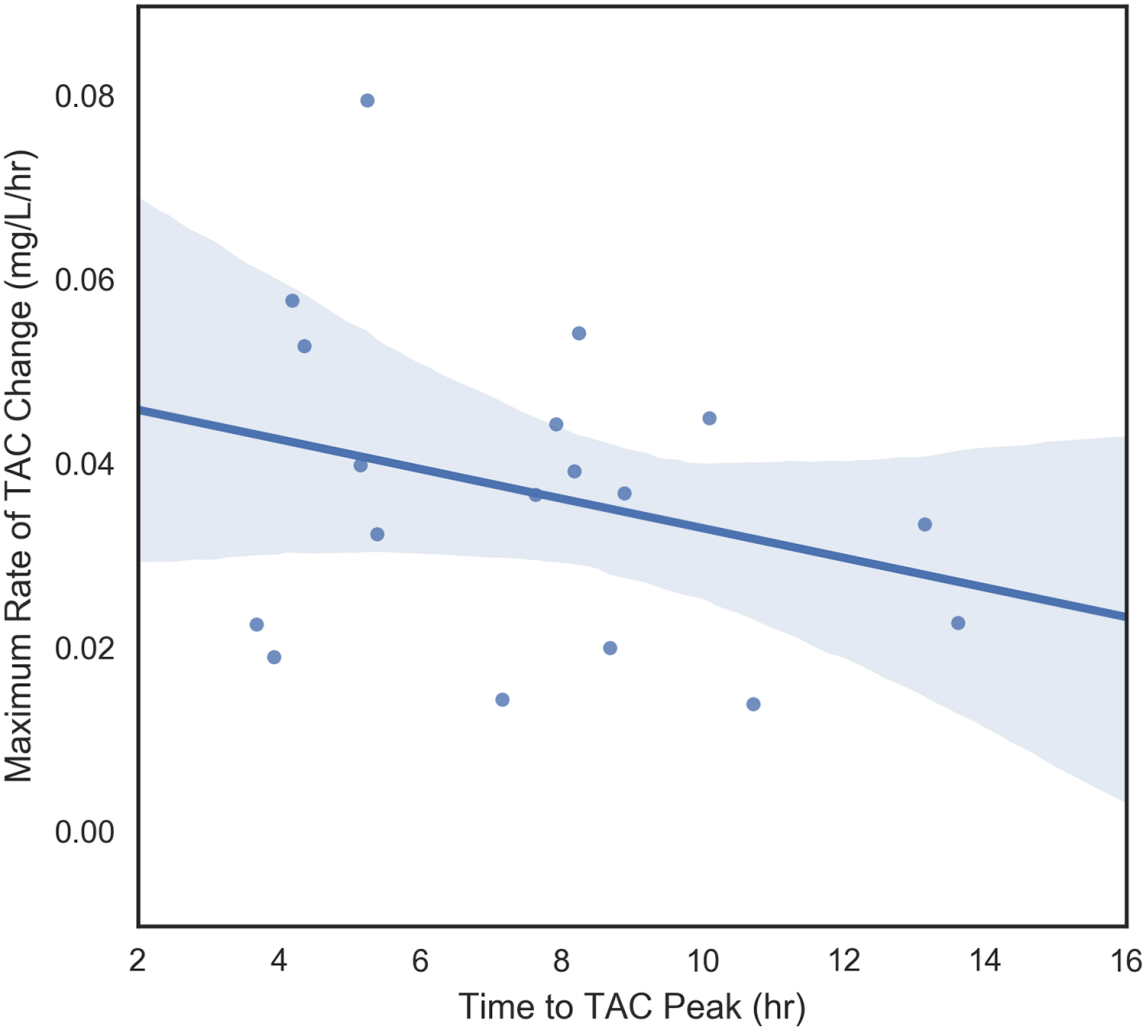
Time to TAC peak vs maximum TAC rate.

**Fig 12:**
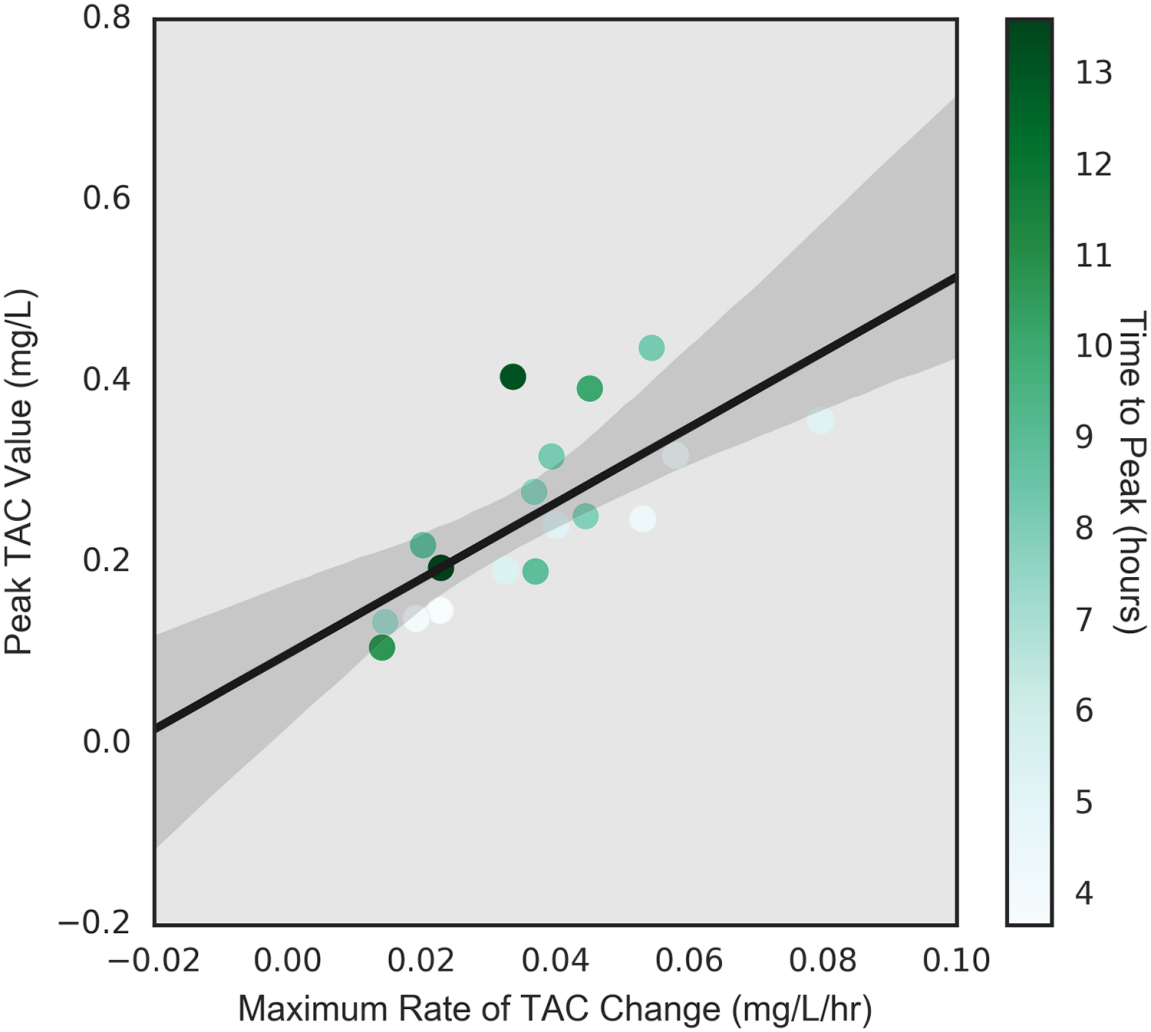
Relationship of time to peak, peak TAC, and maximum rate of TAC.

Figs 9 through 11, examine the relationships between time to peak TAC and the other three dependent variables (peak TAC, average and maximum rate of TAC increase). While the figures suggest potential trends, none of the simple linear model fits were significant at the alpha = 0.05 level. The strongest relationship was found between the average rate of TAC increase and time to peak TAC (*p* = 0.073) with an R-square of 0.187 (*r* = 0.43). Fig 12 shows the inter-relationship of peak TAC, maximum TAC rate, and time to peak TAC. We observe that lowest peak TAC value correspndes to the lowest maximum rate of consumption, but has one of the longer time to peaks. We also observe that the person with the longest time to peak, has the second highest peak TAC value, but a more or less middle rate of consumption. The point here being that the time to peak is related to multiple factors including drinking pattern and length of drinking episode.

Through the rest of this paper these four dependent variables are considered. These analyses show that the different measurements of rates, measure similar concepts; the rate of alcohol consumption significantly affects the peak blood alcohol concentration (both statistically and in terms of the amount of variance explained); and time to peak is not particularly related to maximum blood alcohol concentration, nor rate of drinking.

#### Categorical findings

Boxplots and ANOVA analyses were run on categorical variables versus the dependent variables: 1) peak TAC value, 2) average rate of TAC change prior to peak, 3) maximum rate of change of TAC prior to peak, and 4) time to peak TAC value. As an exploratory data activity, issues of multiple comparisons were not considered. The relationships with the p-values of 0.05 or smaller as assigned by ANOVA tests are shown in Table 1.

**Table 1:** Association of dependent variables with ordinal or categorical variables.

Dependent Variable	Independent Variable	Effect Size (eta squared)	P-value
Peak TAC Value	Post: Drank More than Intended	0.43	0.0032
	AUDIT: How Often 5 or more drinks at one occasion	0.42	0.049
	AUDIT: How Many Drinks per Typical Day	0.42	0.049
Average Rate of TAC Increase	QF: How Often Drink Any	0.82	0.00038
	Post: Drank More than Intended	0.49	0.0012
	QF: How Often Drink Beer	0.81	0.0018
	AUDIT: How Often Have Drink	0.57	0.0067
	QF: How Often Drink Wine	0.68	0.0092
Maximum Rate of Change of TAC	Post: Drank More than Intended	0.50	0.00096
	AUDIT: How Often Have Drink	0.51	0.017
	QF: How Often Drink Beer	0.66	0.031
	QF: How Often Drink Wine	0.59	0.035
	QF: How Often Drink Any	0.59	0.037
Time to TAC Peak	QF: How Often Drunk	0.59	0.014
	Post: Time Moving Quickly	0.29	0.021
	Plans: Other Places	0.37	0.030
	Plans: Travel Taxi	0.24	0.040

The response on the next morning post survey of having drunk more than planned was very highly associated with peak TAC level, average rate of TAC increase, and maximum rate of TAC increase. Higher drinking rates and drinking volumes were associated with several components of the AUDIT Scale. Patterns of drinking beer and wine specifically were significantly associated with the rate of consumption, though not with the peak TAC level. The time to reach the peak TAC level was associated with several different metrics. Participants who planned to travel by Uber or a Taxi reached their peak TAC level significantly faster than those who did not plan to do so. Participants with a shorter time to peak TAC significantly reported “Yes” to “Did time pass much more quickly than expected (did you lose track of time)?”

#### Top simple regression findings

Comparisons of pairwise continuous variables using simple linear regression with significance better than 0.05 are shown in Table 2. Again, it should be noted that this was a data mining activity. Total number of drinks consumed throughout the crawl was significantly related to both the total amount of money spent on alcohol and one’s AUDIT score. The total amount spent on alcohol was significantly associated to grade point average as well as the AUDIT score. Individuals with higher AUDIT scores reported drinking for a longer time (more time elapsed during the event). Lastly, number of missing surveys was significantly associated with time to peak TAC.

**Table 2:** Regression results of pairwise continuous variables.

Variable 1	Variable 2	R-square	P-value
Total Spent on Alcohol	Total Number of Drinks	0.52	0.00073
AUDIT score	Total Number of Drinks	0.43	0.0030
GPA	Total Spent on Alcohol	0.29	0.022
Number of Missing Surveys	Time to Peak TAC	0.25	0.035
AUDIT score	Elapsed Time of Event	0.23	0.042
AUDIT score	Total Spent on Alcohol	0.23	0.043

#### Analysis by period

**Fig 13:**
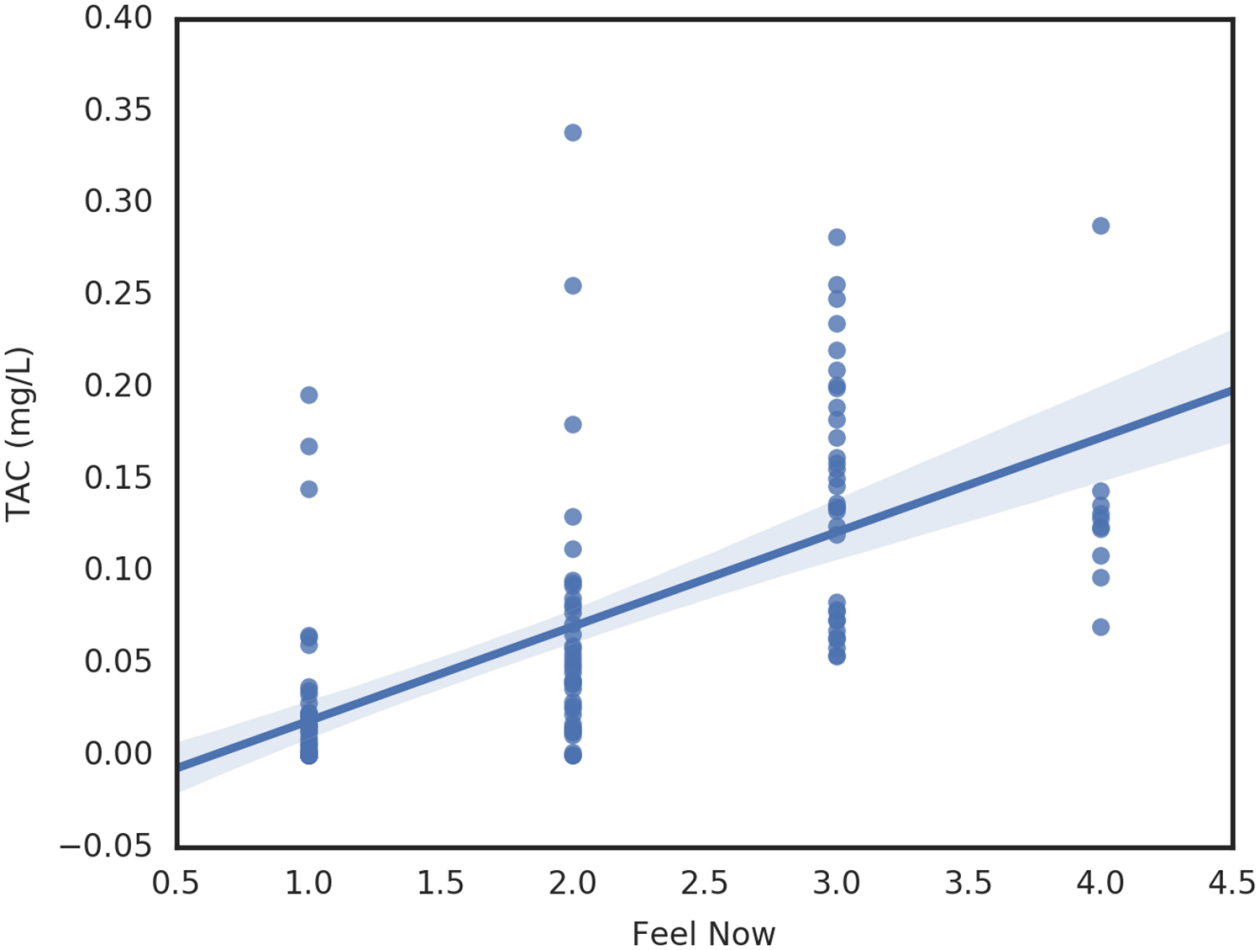
Relationship between TAC value and Feels Now.

For the analysis by period the smoothed TAC value at the period’s survey time and the instantaneous rate of TAC change (Savitzky-Golay derivative) at the survey time were taken to be the dependent variables. These were regressed against key continuous variables collected each survey period. Relationships that are significant at better than 0.05 follow in Table 3. Of particular interest is the relationship between TAC and subjective intoxication. The plot of TAC versus Feel Now is shown in Fig 13. We note the wide variance in the Feel Now values and further note that we have repeated measures for each individual which we need to account for in the analysis.

**Table 3:** Regression results for analyzes by period.

Dependent Variable	Independent Variable	R-square	p-value
Rate of TAC	Number of Drinks	0.044	0.0058
TAC Value	Feel Now	0.43	6.99E-19
TAC Value	Number of Drinks Purchased	0.12	2.50E-05
TAC Value	Number of Drinks	0.069	0.00053
TAC Value	Money Spent on Alcohol	0.066	0.0024

**Fig 14:**
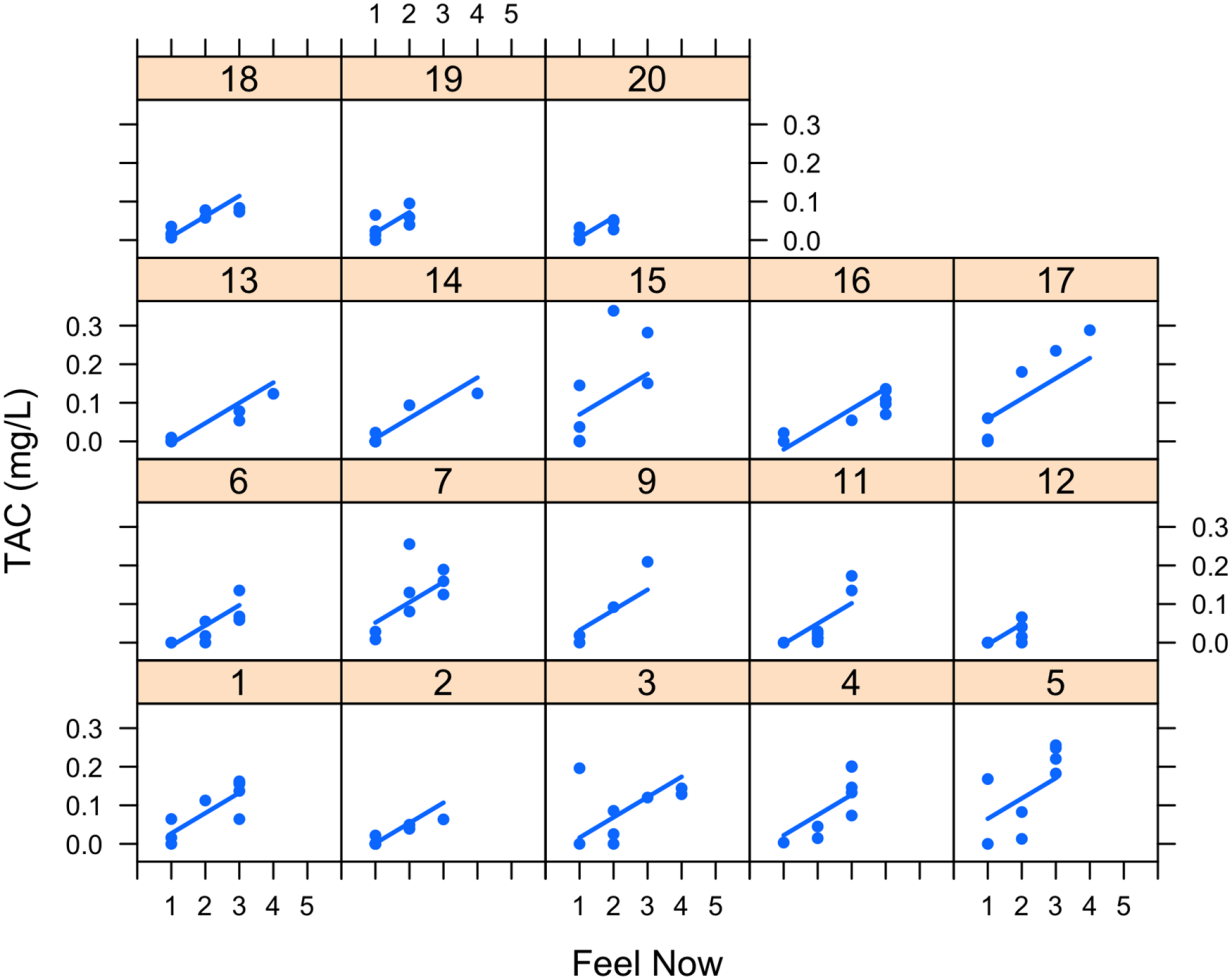
TAC versus Feel Now with participant as a random effect. Participant number is in the header of each subplot.

Fig 14 is a lattice plot of the Feel Now data for each participant. We observe that the data are consistent with the slope for each participant being the same statistically, but the data support fitting different intercepts. The interclass correlation coefficient (ICC) is 0.163 indicating that Participant explains roughly 16% of the variation in TAC. Several mixed-effect models were fit with Participant as a random effect. Feel Now was highly significant (p < 0.00001) when added as a single fixed-effect. Theory suggested that the participant’s AUDIT score might also influence TAC level, but the AUDIT score was not significant either when entered alone or with Feel Now. The mixed-effects model with Feel Now and AUDIT score had worse AIC and BIC scores than a Feel Now linear fixed effect. In all these models, Participant was an intercept random effect only. Attempts to fit Participant as both intercept and slope random effects failed to converge, indicating problems with this model. This is also consistent with the observations previously made about intercepts and slopes in Fig 14.

Finally, as TAC varies over time, time should be controlled for when measuring the relationship between TAC and Feel Now. Further, in the study period the relationship between TAC and time was not linear, rather it rose at the beginning of the event and fell later. While complex models could be fit to this, for the framework of a mixed-effect model, we used a quadratic model for time. The model used is

$$TAC_{i}=\beta_{0}+\beta_{1}*Feel\;Now_{i}+\beta_{2}*T_{i}+\beta_{3}*T_{i}{}^{2}+\gamma *Participant\_Num_{i}+\epsilon_{i}$$

where i is the observation number, *TAC*_*i*_ is the TAC value processed as described above, *Feel Now*_*i*_ is the associated value from the Feel Now survey item, *T*_*i*_ is the time at which the TAC value and associated Feel Now survey are taken, *Participant_Num*_*i*_ is the identification number of each participant entered as a random effect, and *ϵ*_*i*_ is the residual error. The *β* portion of this model represents the fixed effects.

The AIC, BIC, and log Likelihood fit statistics (-425.20, -407.73, and 218.60, respectively) are all better than those for a mixed-effect model without Time (-398.74, -387.09, 203.37). Of the models explored this model has the smallest residual of 0.0434. (The residual is 0.0496 for the mixed-effect model without Time.) Both Time and Feel Now variables are very highly significant. The summary table from this analysis is shown in Table 4.

**Table 4:** Summary of mixed-effect modeling of TAC versus Feel Now and time with participant as a random effect.

Random Effects:	Formula:	~1 | Participant_num			
	Intercept	Residual			
Std Dev	0.0328	0.0434			
Fixed Effects:	SMTACSG ~ Feel Now + Time + Time^2				
	Value	Std. Error	DF	t-value	p-value
(Intercept)	-0.037	0.013	115	-2.954	0.004
Feel Now	0.027	0.006	115	4.436	0.000
Time	0.020	0.004	115	4.630	0.000
Time^2	-0.001	0.000	115	-2.827	0.006

#### Plans versus actual

**Fig 15:**
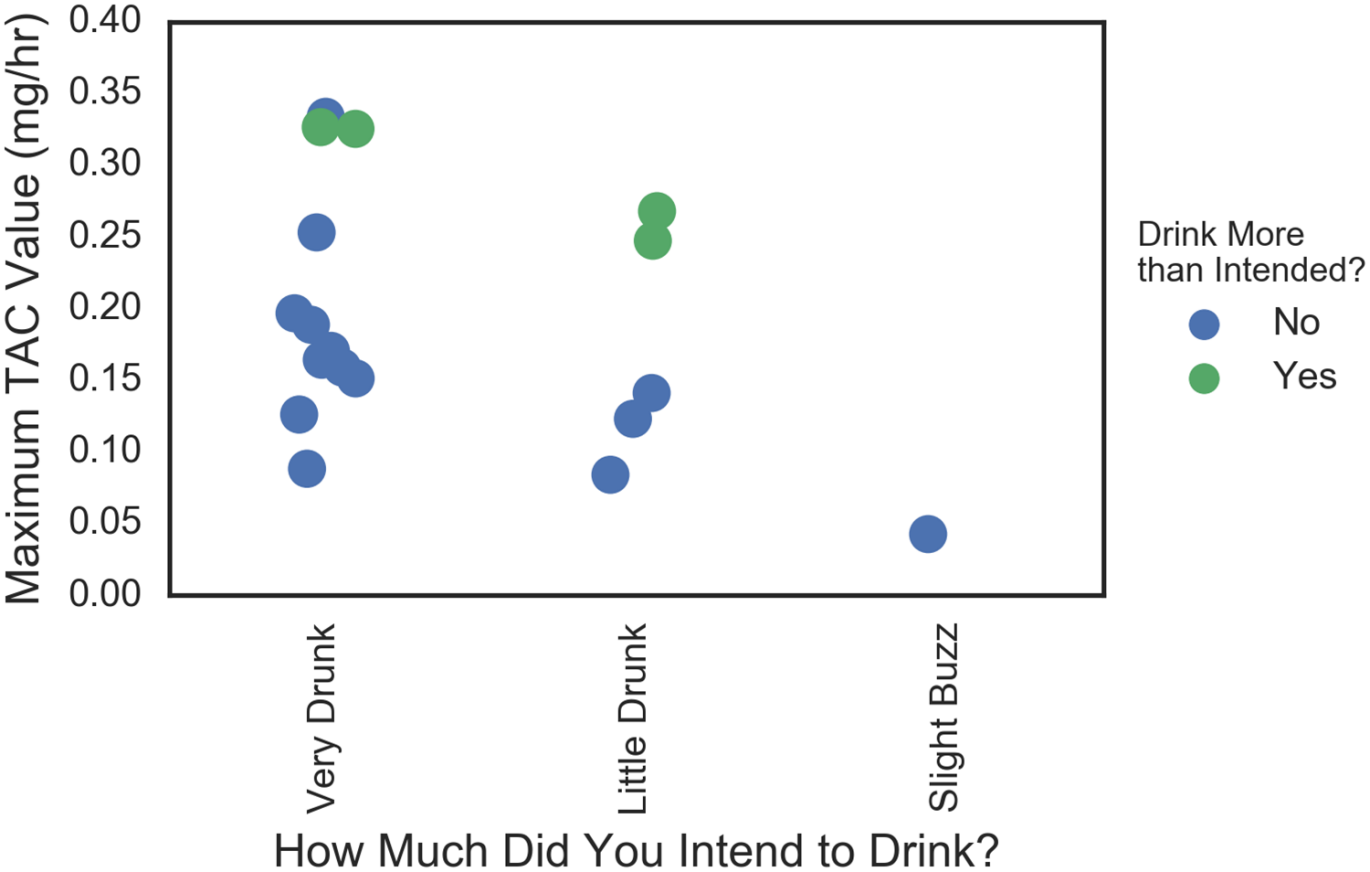
Plans to drink versus actual in terms of Peak TAC. Data are jittered for visibility.

The relationship between how much people intended to drink (measured prior to the crawl), whether they drank more than intended (measured post-crawl), and their actual peak TAC values was explored. These data are plotted in Fig 15 where we observe that those who drank more than intended were, with one exception, the participants with the highest TAC value within their “planned drinking” group. Further, again with one exception there was separation between the “More than intended” and the rest in each planned drinking group.

**Fig 16:**
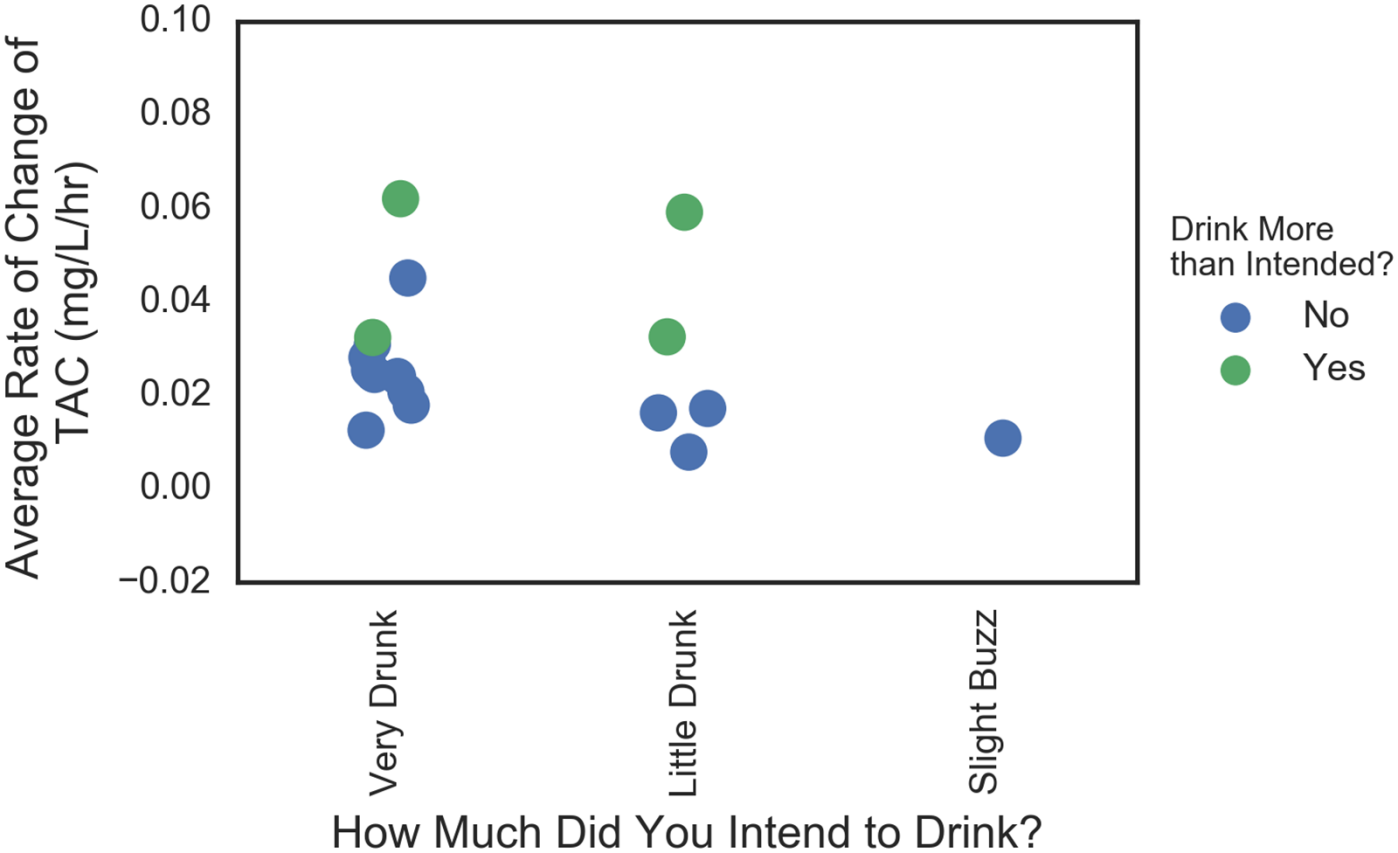
Plans to drink versus actual in terms of average TAC rate. Data are jittered for visibility.

**Fig 17:**
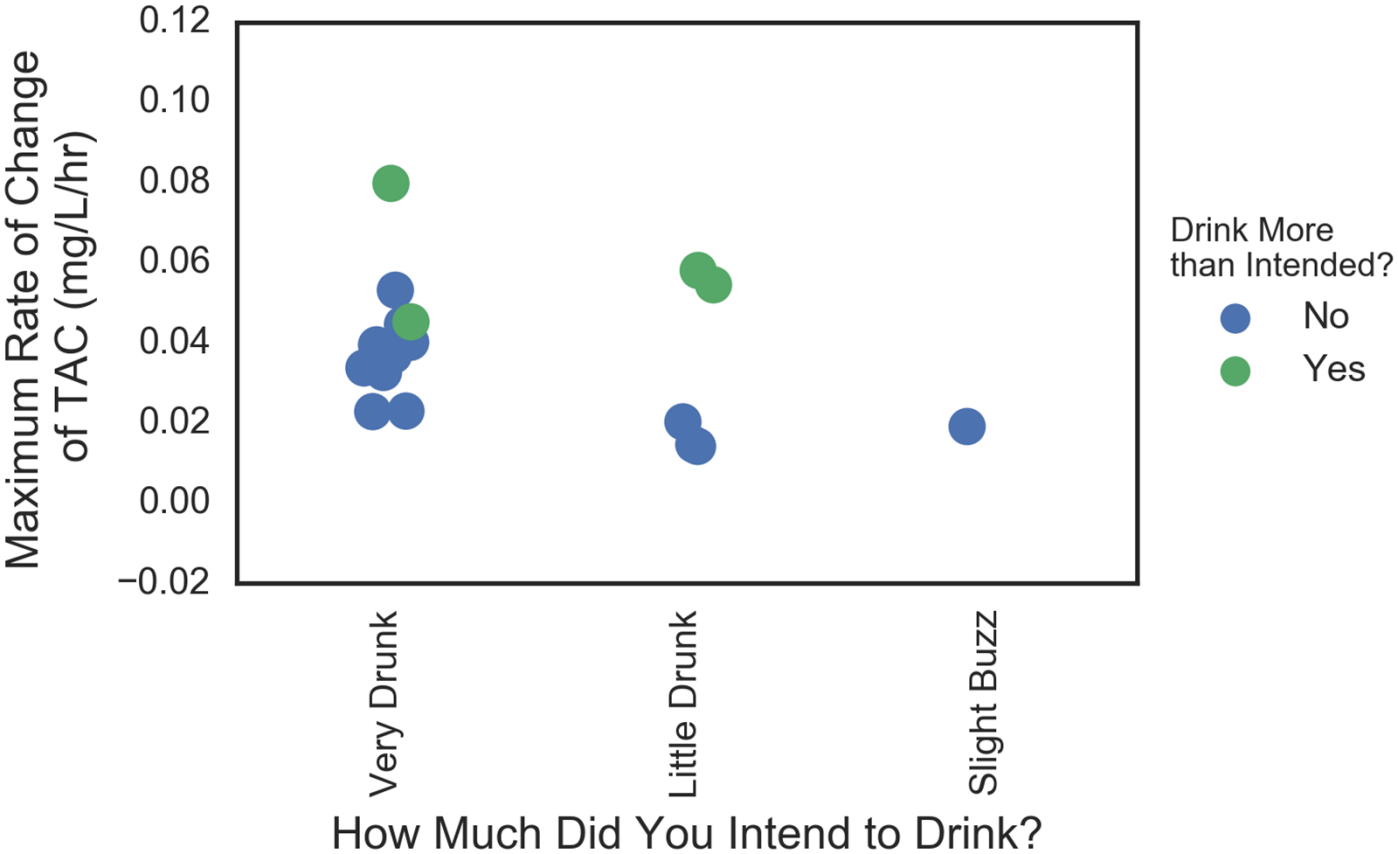
Plans to drink versus actual in terms of maximum TAC rate. Data are jittered for visibility.

This analysis was repeated on the rate of TAC change which was a proxy for the rate of consumption. Figs 16 and 17 show similar plots for the average and maximum instantaneous rates of change. The story is similar in that the high consumption rate values corresponded with drinking more than intended (again with one exception of some who drank rapidly and not more than intended). There was less separation between those that drank more than intended among those who planned to get “Very Drunk” and those that did not.

## Discussion

### Data visualization

There is a great need for more event-level studies and *in situ* explorations of drinking behavior [2 30]. As illustrated here, event-level data can be quite complex. Further, the dynamical nature of drinking events is often difficult to analytically and graphically represent. The present study explores potential approaches to examining drinking events using a multi-method, “big data, small n” framework.

From a data management perspective, data such as these can be hard to reconcile as co-occurring data may reside in separate data sets, formats may not be conducive to comparison (e.g., time information contained in a time variable in some cases, and coded in variable names in others), and time scales may need alignment, calibration, or correction. Data visualization can greatly assist in integrating data sets and understanding the temporal story captured by data collected over the course of an event. We believe the “baseball card” style presentation provides a nice descriptive visualization that allows researchers to view multiple types of data at one time. Similarly, viewing geospatial data for multiple participants during an event givens researchers a sense of mobility during drinking events. When paired with TAC data, EMA data and the like, these data may eventually be useful to identify leverage points [22] for real-time interventions could feasibly be implemented.

Beyond, pilot testing methodological and visualization approaches, this paper also sought to examine some exploratory aspects related to drinking events using data that are seldom available in drinking event studies.

### Exploratory findings

#### Main TAC findings

In this study, peak TAC was significantly related to both the average and maximum rate of TAC increase and the maximum rate of TAC increase was significantly related to average rate of TAC. It is clear in Figs 6 to 8 that the maximum rate of TAC was much more highly correlated with peak TAC than the average. Though a high correlation was expected, we can be confident that these measures were not equivalent. For example, individuals who consume alcohol at a fairly steady rate, will have a maximum rate that is very close to the average rate. Nonetheless, some individuals have a noticeable difference between the two. This can be an indication of either a period of drinking more slowly followed by drinking more rapidly, a period of drinking more rapidly followed by drinking more slowly, or something more complicated. Though no significant relationships were found between time to peak TAC and the other dependent variables in this case, the strongest (yet not significant) relationship between average rate of TAC increase and time to peak TAC should be further explored. A relationship was expected to emerge as time to peak TAC was used to derive the average rate of TAC, but there were other sources of variance involved in this metric. In particular, the length of time one drinks does not immediately imply neither rapid or slow consumption of alcohol nor the quantities of alcohol that were consumed at any given time.

#### Categorical findings

In the categorical explorations, findings (i.e., relationships between typical drinking behavior and AUDIT scores) were consistent with past research [45] and likely some other known associations would show up as significant if these tests had more power and the test assumptions were better met. Interestingly, regularly consuming beer or wine in the weeks prior to the bar crawl was significantly associated with the rate of consumption during the drinking event. It is not clear why this association would exist. It is also intriguing that participants who had a safe transport home (Uber or Taxi) at the end of the event reached their peak level of intoxication earlier in the night. These individuals may have felt more comfortable consuming drinks quickly since they were not concerned with the risks associated with driving drunk later in the evening. Not surprisingly, participants who reached their peak intoxication earlier in the crawl felt that time passed much more quickly throughout the evening. It would be beneficial to continue to explore these possible event-level associations.

#### Regression findings

In the pairwise regression results, several variables were related to the known AUDIT score and several others that were expected to be related did correlate (i.e., amount of money spent was correlated to the total number of drinks purchased). It is expected that individuals with higher AUDIT scores would likely consume more drinks during a drinking event than those with lower scores [46]. Nothing particular novel or interesting was observed by this analysis. More interesting findings were found for the four dependent variables of interest (discussed previously).

#### Analysis by period

At each survey period, rate of TAC change was significantly related to the number of drinks consumed and the TAC value was significantly associated with subjective intoxication (Feel Now), number of drinks purchased, number of drinks consumed, and the amount of money spent on alcohol. Most of these relationships were expected or were already observed in the participant level analysis. The one finding of interest is the correlation between TAC value and the self-reported Feel Now scale. This single item has been utilized in past research [15 16 39 40] but it is not yet clear how well people are able to subjectively sense their level of intoxication. In a recent field study, people were more able to assess their intoxication at lower blood alcohol content but there was a point at which the addition of extra drinks was no longer detectable [47]. In this study, the relationship between Feel Now and TAC varies by time and this suggests that ability to assess TAC level may start out well but deterorate as the night progresses. The relationship between TAC values and subjective intoxication in real time should be further explored especially given the importance between self-assessing intoxication and decisions to drive after drinking.

#### Plans versus actual

Another relationship to further explore is the association between how much people intended to drink as measured on the pre-event survey, whether they drank more than intended as measured by the post event survey, and their actual peak TAC values as measured by the SCRAM device. While the numbers of observations were small in this study and methods were exploratory, these relationships should be followed up in subsequent studies to see to what extent those who drank more than intended also have the highest TAC value within their planned drinking group and to what degree the distribution of TAC for each planned drinking group is bi-modal. If either of these observations hold in confirmatory studies, they could be used to develop thresholds for intervention strategies such as communicating to the participant when they are at risk of drinking more than intended.

### Limitations

Given the complex nature of this data and the varying data collection methods, it is not surprising that many complications were experienced. Missing surveys resulted in a number of issues. All individuals completed surveys, but some failed to complete the surveys at one or more points in time. This leads to noise in a number of metrics such as how many drinks participants had or what types of drinks they consumed. Analysis of missing data (explored in a manuscript that is in progress) suggested that it may be possible to predict participants who are likely to be poor respondents and strategies could be implemented to reduce this problem. Even when surveys were completed, some of the items were limited in their wording. For instance, the questions concerning how many drinks were purchased at each survey point and how much money was spent were ambiguous. Purchases could have been for others at the bar and not the participant. It would be better to ask how much was spent or how many drinks were purchase for yourself.

In this study, geospatial data were also problematic. GPS data were only collected when an EMA survey was completed, therefore, there could have been a substantial amount of time that elapsed between data points. In addition, since GPS data were only collected at survey times, the data are too coarse to do much analysis. Movement to individual bars or the length of stay in particular bars was not available. From the surveys, we know which bars were actually visited, but not the order, route, or timing. Observe on the baseball card plot for Participant 1 (Fig 1) that the 21:00 survey is missing. Hence we have a six-hour period of missing movement (as evidenced in Fig 3), which is a substantial amount of time. After the first portion of the bar crawl, surveys were only collected in three hour windows. This inconsistent length of time between surveys resulted in a large amount of unknown data. For example, participant 14 claims to have gone to 5 bars, but we are missing those visits because the GPS data were collected 3 hours apart in the evening (i.e. only at survey completion times). The three hour windows were probably too long and a lot of activity happened within that period that is hard to resolve. If one of these surveys is missed, it represents a significant loss of data. Consistent time windows also facilitate comparisons of one period to another. It is also possible that participant’s memory over three hours is less accurate than over one. Future studies might examine using real-time GPS tracking to mitigate some of these issues.

This pilot study had a small sample size of *n* = 20, which dropped to 18 and 16 for some of the analyses. Individual subsets of the data were even smaller. Therefore, there was limited power to observe patterns that may exist and in some cases groups were too small for meaningful statistical estimation. Also it is important to note, given the pilot nature of the study, we used exploratory data mining methods without regard for multiple comparisons. Thus all relationships found and reported here are intended to develop hypotheses that can be examined in confirmatory studies.

While the use of transdermal data is a substantial improvement over other objective biological measures of intoxication, there were still caveats. The transdermal data provides a dynamical look at TAC but we are still not able to tell how rapidly drinks may have been consumed during an event. This specific behavior may only be quantifiable when the participant is actually observed drinking in real time. Furthermore, the lag between initial alcohol consumption and TAC detection is estimated at 45 minutes; there is undoubtedly subject-to-subject variance. A recent study found that the time-lag of alcohol appearing transdermally may be increased at higher doses of alcohol [48]. These sources of variance were not feasible to study during this project and it would be useful for future studies to quantify them.

### Conclusions and future directions

In conclusion, this study examined potential methods to measure and analyze drinking event data. Further, we add to the extant body of literature related to dinking events by identifying some potentially interesting areas for future research. As smart technology becomes a viable option for real-time interventions, studies capturing the complexity and dynamical nature of *in situ* drinking behavior will become increasingly valuable. Such studies can be used to tune dynamical models and simulations, test real-time interventions, and better understand how alcohol consumption in various contexts results in problematic outcomes. Future studies are needed to refine the methods used here.

## Supporting information

S1 Dataset Bar crawl 2016 dataset. (XLSX)
